# Antimicrobial consumption in food-producing animals in Thailand between 2017 and 2019: The analysis of national importation and production data

**DOI:** 10.1371/journal.pone.0283819

**Published:** 2023-04-27

**Authors:** Angkana Lekagul, Supapat Kirivan, Natthasit Tansakul, Charunee Krisanaphan, Julaporn Srinha, Thitiporn Laoprasert, Wanwisa Kaewkhankhaeng, Viroj Tangcharoensathien

**Affiliations:** 1 International Health Policy Program, Ministry of Public Health, Nonthaburi, Thailand; 2 Department of Pharmacology, Faculty of Veterinary Medicine, Kasetsart University, Bangkok, Thailand; 3 Food and Drug Administration, Ministry of Public Health, Nonthaburi, Thailand; 4 Department of Livestock Development, Ministry of Agriculture and Cooperative, Bangkok, Thailand; 5 Department of Fisheries, Ministry of Agriculture and Cooperative, Bangkok, Thailand; University of Ilorin, NIGERIA

## Abstract

Antimicrobial resistance (AMR) threatens health security and the economy worldwide. AMR bacteria can spread across humans, animals, food webs and the environment. Excessive use of antimicrobials in food-producing animals has been recognised as one of the main drivers of the emergence of resistant bacteria. This study aims to quantify and identify patterns of antimicrobial consumption in food-producing animals in Thailand in a three-year period (2017–2019). Milligrams of active ingredient from total volume of imported and locally manufactured products minus exports were obtained from Thai FDA. Annual population production of food-producing animals in 2017, 2018 and 2019 was compiled and validated through cooperation between the Department of Livestock Development (DLD), Department of Fisheries (DOF). The total amount of antimicrobial consumption for food-producing animals decreased 49.0% over the three-year period from 658.7 mg/PCU_Thailand_ in 2017 to 336.3 mg/PCU_Thailand_ in 2019. In 2017, the most common antimicrobials used was macrolides which was replaced by aminopenicillins and pleuromutilins in 2019, while tetracyclines was consistently common over the three-year period. Consumption of the WHO Critically Important Antimicrobials (CIA) group declined significantly over this period, from 259.0 in 2017 to 193.2 mg/PCU_Thailand_ in 2019 (a 25.4% reduction). Findings from this study were in line with national policies which curtails prudent use of antimicrobials in food-producing animals. The government should maintain the decreasing trend of consumption, in particular of the CIA category. Improving information systems which captures consumption by specific species contributes to precision of interventions to minimise prudent use in each species.

## Introduction

Antimicrobial resistance (AMR) is one of the most critical health security threats and undermines the economy worldwide. AMR bacteria can spread across humans, animals, food chain and the environment. Resistant bacteria can be transmitted to consumers when they consume inadequately cooked AMR-contaminated meat products, or farmers have direct contact with animals and their environment [1].

The excessive use of antimicrobials in food-producing animals has been recognised as one of the main drivers of the emergence of resistant bacteria [2]. Apart from using antimicrobials for treatment of infectious disease, it is a common practice to use antimicrobials in food animals to control and prevent the spread of infection or disease and antimicrobials are used to promote the growth of livestock in many countries. Of 112 World Organisation for Animal Health (WOAH) member countries, 26% reported the use of antibiotic growth promoters in 2019 [3].

To tackle AMR, international organizations recommend several implementation strategies for both human health and agriculture. The Global Action Plan on Antimicrobial Resistance (GAP-AMR) was adopted in 2015 by the World Health Assembly Resolution (WHA68.7) [4]. One of the strategic objectives of GAP-AMR is to “Strengthen the knowledge and evidence base through surveillance and research”; among others, the Resolution urges World Health Organisation (WHO) and WOAH member states to collect and report data on use of antimicrobial agents in humans and animals [5]. In animals, monitoring antimicrobial consumption is essential in contributing to understanding the situation of antimicrobial usage in countries so that trends can be monitored and outcomes and impact of optimising use of antimicrobials policies can be assessed.

In human health sector, there has been significant progress in the monitoring of antimicrobial use and consumption; however, the progress in the animal health sector has lagged behind [6]. Of 160 countries that responded to the WOAH questionnaire, only 133 countries provided quantities of antimicrobial agents, of which eight countries provided quantitative data for more than one year between 2017 and 2019 [3]. In European countries, antimicrobial consumption in animals is reported annually by the European Medicines Agency established the European Surveillance of Veterinary Antimicrobial Consumption (ESVAC) project since 2009 [7].

Global consumption of antibiotics in food animal production was estimated at 93,309 tonnes of active ingredient in 2017. Moreover, based on the continued rise in global demand for livestock products, global antibiotics consumption by livestock sector is predicted to increase about 11.5% in 2030 [8]. In the latest 2021 report, the volume of antimicrobial consumption in food-producing animals in 31 European countries was 84.4 mg/ population correction unit (PCU) (5,219.6 tonnes) [9]. In Asia, a few countries reported the antimicrobial consumption in food producing animals at the national level. For example, in Japan, 447 tonnes of active ingredient were used in pigs in 2018 [10].

Since 2016, the surveillance of human and veterinary antimicrobial consumption in Thailand has been established by collaboration among One Health partners which includes Food and Drug Administration (FDA), Department of Livestock Development (DLD), Department of Fisheries (DOF), international partners such as WHO, Food and Agriculture Organisation (FAO), United States Agency for International Development (USAID), private sector and academia [11]. The data source for the Surveillance of Antimicrobial Consumption in Thailand was based on the mandatory annual reporting system of total volume of imported and manufactured medicines including antimicrobials by all Market Authorization Holders (MAHs). These reports were submitted to and verified by the Thai FDA according to Article 85 of the Drug Act B.E. 2510 1967). Thailand is one of the leading livestock production countries. In 2022, about 10 million fattening pigs, 300 million broiler chickens, 65 million layer chickens, 8.5 million broiler ducks, 17 million layer ducks, and 10 million cattle were produced in Thailand [12].

This study aims to quantify and identify patterns of antimicrobial consumption in food-producing animals in Thailand in a three-year period (2017–2019). It contributes to monitoring progress towards Thailand’s National Strategic Plan on AMR (2017–2021) goals in which one of the targets is a 30% reduction of antimicrobial consumption in food producing animals between 2017 and 2021.

## Materials and methods

### Data source

#### Antimicrobial consumption

National-level antimicrobial consumption was defined as the total volume of annual imported and locally manufactured veterinary antimicrobials minus total exportation. According to Drug Act B.E. 2510 (AD 1967), all pharmaceutical manufacturers and importers are required to submit an annual report to the Thai FDA, consisting of their total volume of production and importation of registered products. These mandatory reports are to be submitted to Thai FDA by 31 March of each following year [13]. In 2020, there were 1,859 veterinary antimicrobials registered in Thailand. There were 35, 53 and 52 total importers and 34, 56 and 54 manufacturers of veterinary antimicrobials who submitted the reports to the Thai FDA in 2017, 2018 and 2019, respectively.

#### Animal population

The number of food-producing animals in 2017, 2018 and 2019 was compiled and verified through cooperation between the Department of Livestock Development (DLD), Department of Fisheries (DOF), private animal production experts and relevant stakeholders, and the final figures were agreed upon by consensus.

This study included four main animal species: pigs, poultry, cattle and aquatic animals. Pigs included breeding and fattening pigs; cattle included beef, dairy and dry cows; poultry included broiler chickens and ducks that are bred and raised specifically for meat production through two main sources: the free market duck by small farm holders and integrated duck production by large manufacturers, and layer chickens and ducks that are raised specifically for the purpose of producing eggs, as well as poultry breeders. The aquatic animals included from both coastal and freshwater sources.

For terrestrial food producing animals, data were collected and verified from three sources: 1) livestock surveys by district and provincial DLD offices, 2) data records from the electronic movement system of DLD, 3) National committees such as egg board and pig board, and 4) large-scale livestock producers. For the aquatic animal population, data were collected from surveys and estimated by DOF. Total annual tonnes of fishes or shrimps were estimated from all provinces having aquaculture which raised major fishes and shrimps in both coastal and freshwater farms.

### Inclusion of antimicrobials in the monitoring system

All veterinary medicines registered with the Thai FDA were assigned an Anatomical Therapeutic Chemical classification system for veterinary medicinal products code (ATCvet code). For the scope of veterinary target antimicrobials, Thailand SAC covered four groups of target antimicrobials as recommended by the World Organisation for Animal Health (WOAH) and European Surveillance of Veterinary Antimicrobial Consumption (ESVAC) [14] (Table 1).

**Table 1:** The scope of target antimicrobials intended for use in food-producing animals.

Target veterinary antimicrobials	ATCvet codes
1. Antimicrobial agents for intestinal use	
Antibiotics	QA07AA
Sulfonamides	QA07AB
Other intestinal anti-infectives	QA07AX
2. Antimicrobial agents for intrauterine use	
Antibiotics	QG01AA, QG01BA
Sulfonamides	QG01AE, QG01BE
Antibacterials	QG51AA
Anti-infectives for intrauterine use	QG51AG
3. Antimicrobial agents for systemic use	QJ01
4. Antimicrobial agents for intramammary use	QJ51

### Data analysis

#### Antimicrobial consumption measurement

Data of imported and locally manufactured veterinary antimicrobials were derived from an electronic database of annual report system operated by the Thai FDA, checked for completeness and verified data with MAHs. Data compilation from registration database included name of active ingredients, strength (amount of active ingredient), and the ATCvet code. Volume of active ingredients was calculated by multiplying the strength of each antimicrobial in the registration database with the amount of finished product reported in the annual report system. Consumption was calculated in milligrams of active ingredient classified by ATCvet classification system and WHO Critically Important Antimicrobials for human medicine (CIA) [15]. Descriptive statistical analyses were performed using the Stata/SE 15 software.

As data captured by the Thai FDA included total volume of imported and locally manufactured products, it was assumed that total imported and local production minus total exports equalled total consumption in a year (assuming that in an efficient market, the stock level remains constant in each year). Though there is no mandate to report exports, the Thai FDA requested all MAHs to provide export volumes of medicines including antimicrobials, and all of them complied with this request.

#### Animal population measurement

Population Correction Unit (PCU), as applied from ESVAC methodology, was used as a denominator for antimicrobial consumption in food-producing animals and was calculated by applying ESVAC reference on Average Weight (AW). According to the ESVAC, PCU is assumed to be a proxy for the animal population at risk of being exposed to antimicrobials [16].

AW is the weight at time of treatment; the AW for each animal species referred to the ESVAC standards. Certain species of food-producing animals in Thailand are not produced by European countries and hence have no AW [14]. Therefore, experts estimated the AW of these animal species including broiler breeder, layer breeder, laying hen, pullet, broiler duck breeder, broiler duck, layer duck, and dry cow. Consensus among experts was reached that AW for these species equals the standing weight (Table 1). For aquatic food animals, fish and shrimp biomasses were estimated from productions and area of aquaculture in the sampled farms [17]. As the PCU methodology was modified from ESVAC by Thailand SAC, it is defined as “PCU_Thailand_”.

## Results

### Animal population

Between 2017 and 2019, the population of the four main species–pigs, poultry, cattle, and aquatic animals–increased. In 2019, the number of pigs was 23,413,075 animals, cattle 6,677,311 animals, poultry 1,876,251,390 animals and aquatic animals 944,095.4 tonnes of biomass. Therefore, the PCU_Thailand_ increased from 6,618,137,577.6 kg in 2017 to 7,632,186,195.8 kg in 2019 (Table 2).

**Table 2:** Food producing animals in the Population Correction Unit (PCU) (2017–2019).

Animal	AW (kg)	Year					
		2017		2018		2019	
		Number	PCU_Thailand_ (kg)	Number	PCU_Thailand_ (kg)	Number	PCU_Thailand_ (kg)
**Pig**		**19,440,682.0**	**1,443,768,505.0**	**24,015,447.0**	**1,770,746,630.0**	**23,413,075.0**	**1,733,877,600.0**
Breeding pigs	240	1,029,281.0	247,027,440.0	1,198,529.0	287,646,960.0	1,211,587.0	290,780,880.0
Fattening pigs	65	18,411,401.0	1,196,741,065.0	22,816,918.0	1,483,099,670.0	22,201,488.0	1,443,096,720.0
**Cattle**		**5,395,012.0**	**2,292,880,100.0**	**6,019,479.0**	**2,558,278,575.0**	**6,677,311.0**	**2,837,857,175.0**
Beef cows	425	4,876,228.0	2,072,396,900.0	5,445,351.0	2,314,274,175.0	6,011,000.0	2,554,675,000.0
Dairy cows	425	245,505.0	104,339,625.0	275,358.0	117,027,150.0	374,607.0	159,207,975.0
Dry cows	425	273,279.0	116,143,575.0	298,770.0	126,977,250.0	291,704.0	123,974,200.0
**Poultry**		**1,793,089,307.0**	**2,085,788,972.6**	**1,860,015,066.0**	**2,113,502,652.1**	**1,876,251,390.0**	**2,116,356,018.3**
Broiler breeders	4	18,100,000.0	72,400,000.0	12,601,103.0	50,404,412.0	17,000,000.0	68,000,000.0
Broiler duck breeders	3.5	321,300.0	1,124,550.0	318,318.0	1,114,113.0	321,342.0	1,124,697.0
Broilers	1	1,594,494,720.0	1,594,494,720.0	1,672,905,728.0	1,672,905,728.0	1,706,363,843.0	1,706,363,843.0
Broiler ducks	3.3	57,207,362.0	188784294.6	46,872,232.0	154678365.6	39,479,236.0	130,281,478.8
Layer ducks	2.5	16,354,585.0	40886462.5	14,489,207.0	36223017.5	15,880,504.0	39,701,260.0
Layer breeders	2	719,900.0	1,439,800.0	546,303.0	1,092,606.0	617,051.0	1,234,102.0
Laying hens	2	55,643,971.0	111,287,942.0	57,322,295.0	114,644,590.0	49,533,033.0	99,066,066.0
Pullets	1.5	50,247,469.0	75,371,203.5	54,959,880.0	82,439,820.0	47,056,381.0	70,584,571.5
**Aquatic animal** *		**795,700.0**	**795,700,000.0**	**867,250.0**	**867,250,000.0**	**944,095.4**	**944,095,402.5**
Coastal aquatic animals	n/a	382,400.0	382,400,000.0	426,575.0	426,575,000.0	457,277.9	457,277,885.9
Freshwater aquatic animals	n/a	413,300.0	413,300,000.0	440,675.0	440,675,000.0	486,817.5	486,817,516.6
**Grand total**			**6,618,137,577.6**	** **	**7,309,777,857.1**	** **	**7,632,186,195.8**

* Metric tonne

### Antimicrobial consumption

#### Overall consumption

The total amounts of antimicrobials for food producing animals were 658.7; 522.0 and 336.3 mg/PCU_Thailand_ in 2017, 2018 and 2019 respectively. The 2019 consumption decreased by 35.6% compared with 2018 and 49.0% compared with 2017 (Table 3). Between 2017 and 2019, the majority of consumption was antibacterials for systemic use (QJ01) (278.3 mg/PCU_Thailand_ in 2019), followed by antimicrobials for intestinal use (QA07) (57.8 mg/PCU_Thailand_ in 2019).

**Table 3:** Antimicrobial consumption (mg/PCU_Thailand_) in food producing animals in Thailand, from 2017 to 2019 (ATC level 3), categorised by Critically important antimicrobials for human medicine [15].

ATC code level 3	Class	Year		
		2017	2018	2019
**Critically Important Antimicrobials**		**259.0**	**289.1**	**193.2**
**QA07A**	Aminoglycosides	5.9	7.8	6.0
	Polymyxins (Colistin)	0.4	23.5	18.6
**QJ01C**	Aminopenicillins	11.4	210.6	125.3
	Aminopenicillins with BLI	0.1	0.1	0.1
**QJ01D**	Cephalosporins (3^rd^ and 4^th^ generation)	0.2	0.3	0.2
**QJ01F**	Macrolides	236.2	36.6	31.9
**QJ01G**	Aminoglycosides	2.7	4.1	4.7
**QJ01M**	Fluoroquinolones	0.6	5.2	5.8
**QJ01X**	Phosphonic acids	1.5	0.9	0.6
**Highly Important Antimicrobials**		**306.0**	**75.4**	**73.0**
**QJ01A**	Tetracyclines	80.3	63.2	62.3
**QJ01C**	Penicillins (non-CIA)	2.0	2.0	2.0
**QJ01E**	Sulfonamides	221.9	6.6	6.3
**QJ01F**	Lincosamides	1.7	3.5	2.4
**Important Antimicrobials**		**20.3**	**76.9**	**55.1**
**QA07A**	Polypeptides	10.5	14.6	18.4
**QJ01X**	Aminocyclitols	2.1	2.0	0.5
	Pleuromutilins	7.7	60.2	36.2
**Antimicrobials currently not used in humans**		**73.5**	**80.7**	**14.9**
**QA07A**	Quinolines	73.3	80.5	14.8
**Total**		**658.7**	**522.0**	**336.3**

Note: data show only API >1 mg/PCU_Thailand_

#### Consumption by antimicrobial class

**Fig 1:**
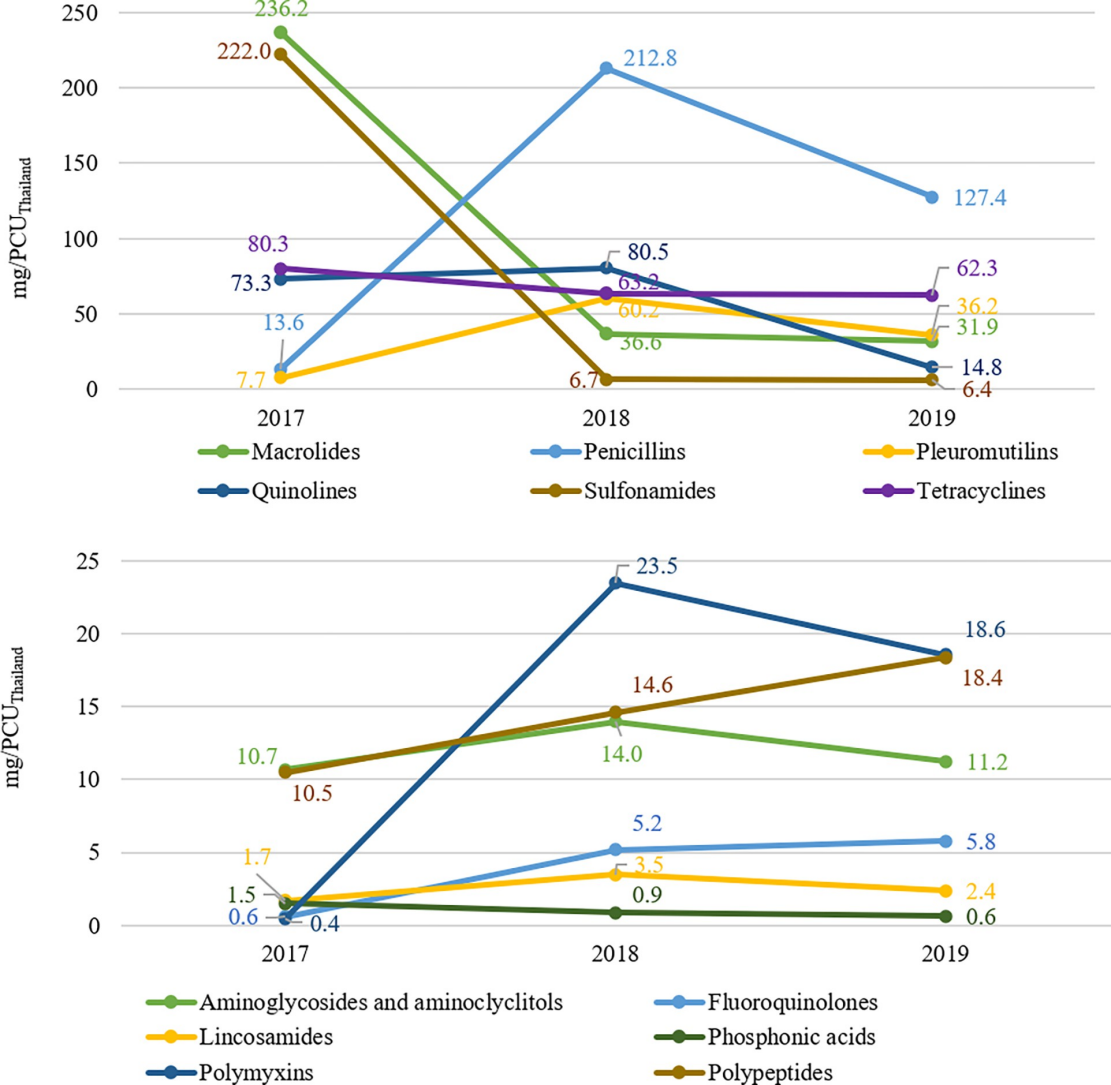
Amount of antimicrobial consumption in food producing animals by antimicrobial class (2017–2019) (mg/PCU_Thailand_). (top) Antimicrobials with >50 mg/PCU_Thailand_ in at least a year between 2017 and 2019. (bottom) Antimicrobials with <50 mg/PCU_Thailand_ between 2017 and 2019 (not included amphenicols, cephalosporins, orthosomycins, phosphoglycolipids).

In 2017, the top three classes of antimicrobials consumed were macrolides (236.2 mg/PCU_Thailand_), sulfonamides (221.9 mg/PCU_Thailand_), and tetracyclines (80.3 mg/PCU_Thailand_); the top three shared more than 80% of total consumption. However, the consumption of both macrolides and sulfonamides decreased sharply in 2018 and 2019. In 2019, the top three were aminopenicillins (125.3 mg/PCU_Thailand_), tetracyclines (62.3 mg/PCU_Thailand_) and pleuromutilins (36.2 mg/PCU_Thailand_). Aminopenicillin group was found to rapidly increase from 11.4 in 2017 to 210.6 mg/PCU_Thailand_ in 2018, but declined to 125.3 mg PCU_Thailand_ in 2019. Among the aminopenicillin group, amoxicillin was the most consumed antimicrobial at 55.3 mg/PCU_Thailand_ in 2019. Usage of other antimicrobial classes such as macrolides, sulfonamides and tetracycline declined each year (Fig 1).

#### Consumption by route of administration

**Fig 2:**
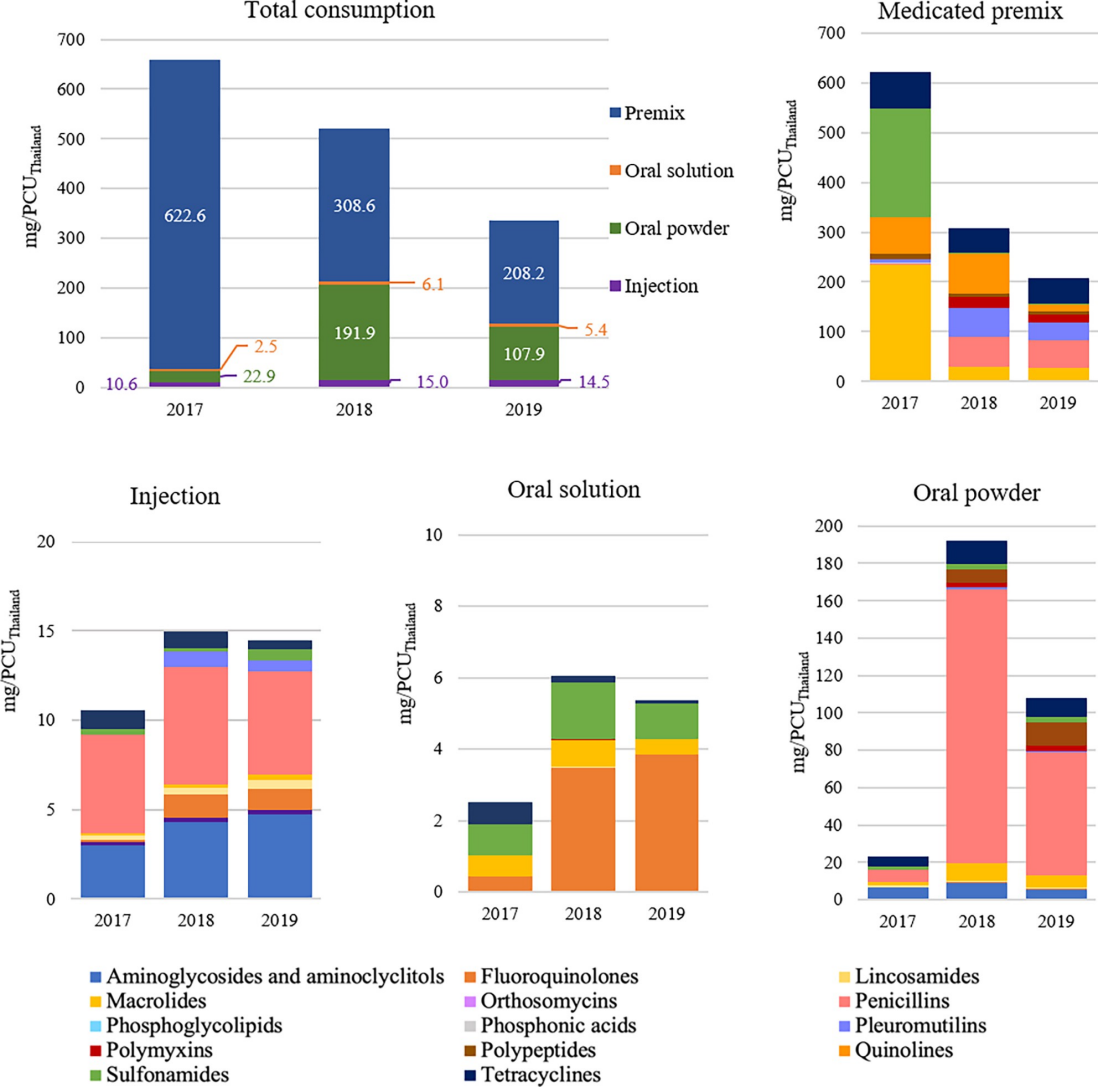
Trends of antimicrobial consumption in food-producing animals by route of administration (2017–2019), total consumption and premix (upper panel), and injection, oral solution and oral power (lower panel) (mg/PCU_Thailand_). Note: Does not include antimicrobials that had less than 1 tonne of consumption (intramammary and other routes).

The most common route of antimicrobial administration was oral administration (Fig 2). In 2017, the main antimicrobial application form was medicated premix at 622.6 mg/PCU_Thailand_ (94.5% of total consumption), followed by oral power (22.9 mg/PCU_Thailand_) and injection (10.6 mg/PCU_Thailand_). However, from 2017 to 2019, the premix form was reduced consecutively from 94.5% to 61.9%, while oral powder form increased from 3.5% to 32.1% (Fig 2).

#### Consumption by CIA groups

**Fig 3:**
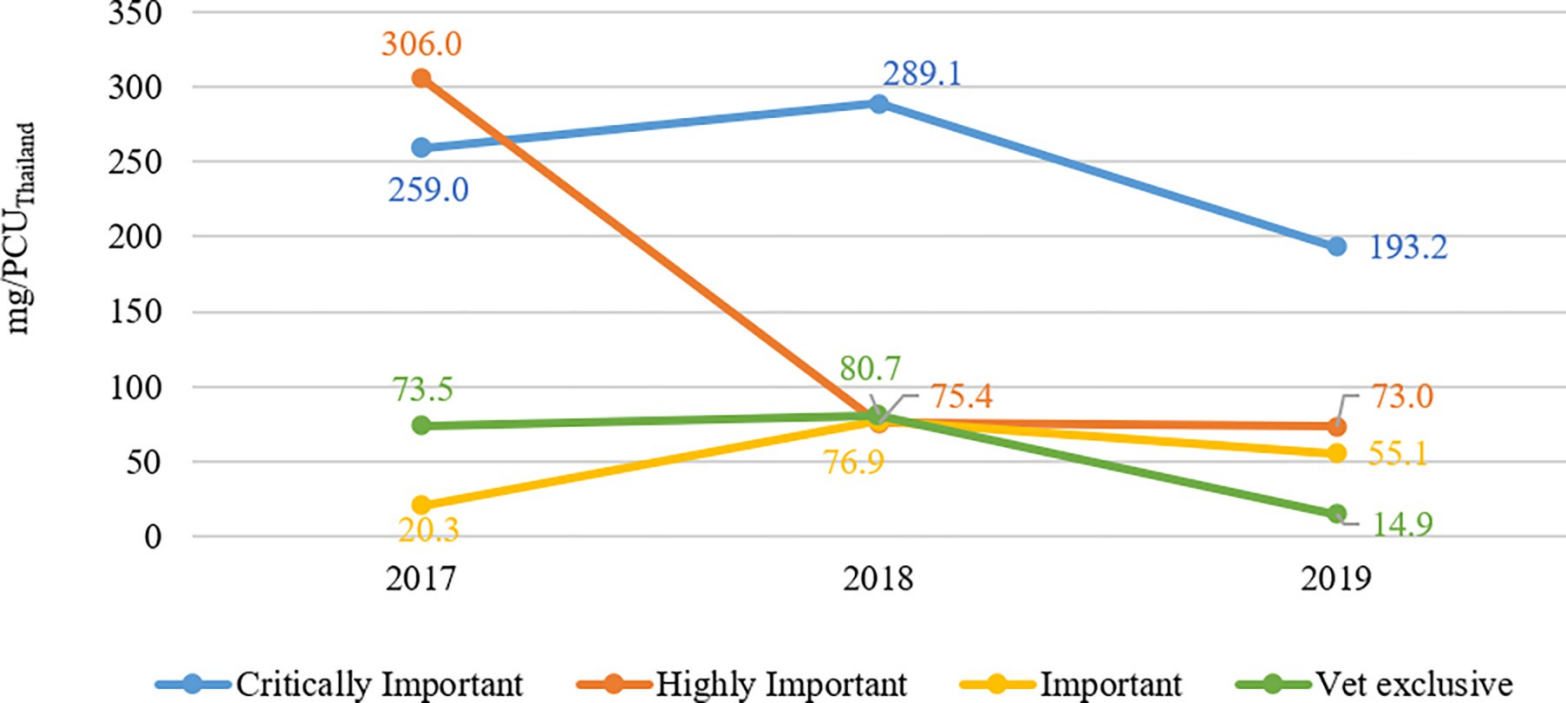
Trends of antimicrobial consumption in food-producing animals by WHO CIA list (2017–2019) (mg/PCU_Thailand_).

Overall, the consumption in food-producing animals in the WHO CIA group declined significantly over the period 2017–2019 (Fig 3). Total consumption of the CIA group in 2019 was 193.2 mg/PCU_Thailand_, 25.4% lower than in 2017 (259.0 mg/PCU_Thailand_). Macrolides was commonly consumed in the CIA group, accounting for 91.2% of total CIA consumption (236.2 mg/PCU_Thailand_) in 2017. The decreasing trend of the CIA group was due to significant reduction of macrolide consumption to 31.9 mg/PCU_Thailand_ in 2019.

Among the highly important antimicrobials in 2017, sulfonamides (221.9 mg/PCU_Thailand_) and tetracyclines (80.3 mg/PCU_Thailand_) were the top two. A significant consumption reduction of highly important category happened from 306.0 in 2017 to 73.0 mg/PCU_Thailand_ in 2019, attributed to 97% reduction of sulfonamides consumption in the last two years (Table 3).

## Discussion

To the extent of our knowledge, this is one of the first studies to assess national level antimicrobial consumption in food producing animals in low and middle-income countries (LMICs). It demonstrated the decreasing trend of antimicrobial consumption in food producing animals in Thailand between 2017 and 2019; by 49.0% from 658.7 in 2017 to 336.3 mg/PCU_Thailand_ in 2019. The level of antimicrobial consumption in food producing animals in 31 European countries in 2021 was 84.4 mg/ population correction unit (PCU) (5,219.6 tonnes) [9]. It is not appropriate to compare our study with European countries due to difference in disease epidemiology, farm biosecurity, source of antimicrobial consumption data, and other contexts.

Macrolides and sulfonamides were the most frequently used antimicrobials in 2017, and aminopenicillins (e.g. amoxicillin, ampicillin) in 2018–2019. This finding was similar to that reported by EU countries, where the top three in 2020 were penicillins (31.1% of total consumption), sulfonamides (9.9%) and macrolides (8.8%) [18]. A similar trend was reported by studies in South-East Asia. A survey of antimicrobial use in livestock in 2019 in Indonesia, Vietnam and Thailand showed that penicillins, tetracyclines, and sulfonamides were the most commonly consumed classes [19]. Similarly, in African countries, tetracyclines was commonly used in animals. A recent survey that investigated frequently used veterinary antibiotics by commercial chicken farmers and pharmaceutical outlets in Africa reported tetracyclines (32.2% of total) and sulfonamides (20.8%) as most commonly used [20]. In Tanzania, data of all veterinary antibiotics records between 2010 and 2017 showed that tetracyclines was consumed the most, accounting for 66.6% of total of 8,090,798 kg [21]. A study in South Africa found that the majority consumptions were of macrolides and pleuromutilins (42.4% of total), tetracyclines (16.7%), sulfonamides (12.4%) and penicillins (10.7%) [22]. A systematic review of antibiotic use in pig production showed that penicillins and tetracyclines were the most commonly used in many countries [23].

In our study, more than half of all antimicrobial consumption in 2019 was of the CIA group (57.4%, 193.2 out of total 336.3 mg/PCU_Thailand_). WHO recommends an overall reduction in use of all classes of medically important antimicrobials (MIA) and complete restriction of routine use of MIA for prevention of infectious diseases that have not yet been clinically diagnosed in food-producing animals [24]. The use of antimicrobials in the CIA category should be limited for treatment purposes with specific indications in animals as it should be last-resort antibiotics normally reserved for the most severe infections in humans.

Appropriate use of antimicrobials can be regulated through classification and requirement such as restricting certain classes such as critically important antimicrobials to prescription-only medicine. Despite these effective interventions, regulations in LMICs focus on the licensing process of medicines [25]. Over-the-counter sale of antimicrobials without prescriptions is the common issue [26]. Thailand totally bans the use of antimicrobial as growth promoter since 2015. Prior to 2018, most veterinary antimicrobials in Thailand were classified as dangerous drugs which do not require a prescription but needs to be dispensed by licensed pharmacists or veterinarians at licensed pharmacies. In 2018, a number of restrictions were imposed on the veterinary use of certain reserved groups. Some CIAs including polymyxins, penicillins, fluoroquinolones and fosfomycins are not allowed to be used for disease prevention through medicated feed. Cephalosporins are prohibited from mixing in feed for all purposes including treatment and prevention [27]. A prescription is required for the sale of quinolones, cephalosporins, macrolides and polymyxin by pharmacies and pharmaceutical companies, and for the sale of all medicated premix (antimicrobials mixed in feed). These regulations came into effect in 2019 [28].

These regulatory measures have resulted in significantly decreased consumption of macrolides (from 236.2 in 2017 to 31.9 mg/PCU _Thailand_ in 2019), and increased consumption of non-CIA class such as pleuromutilins (from 7.7 in 2017 to 36.2 mg/PCU_Thailand_ in 2019). Macrolides are active broad-spectrum antimicrobials used against Gram-positive and Gram-negative bacteria in humans and animals [29]. In swine veterinary practice, macrolides are widely used to control common infections such as enteritis, respiratory infections and arthritis caused by *Streptococcus suis* type 2 in weaned pigs and *Mycoplasma hyosynoviae* in fattening pigs. Furthermore, tylosin, a broad-spectrum macrolide, is mainly used in medicated feed for treatment and prevention of respiratory disease, swine dysentery and porcine proliferative enteropathy [29]. In poultry production, macrolides such as tylosin, tilmicosin are used to treat infections due to Mycoplasma infection and, *P*. *multocida* and *Clostridium perfringens* [30]. However, macrolides are categorised as one of the highest priority WHO CIAs, which are essential for the treatment of specific infections in humans [15]. There have been several reports of high resistance to macrolides in *Enterococcus* in humans [31, 32] and animals [33]. Regulatory measures on restricting use of macrolides achieved significant reduction in 2018 and 2019.

Our study shows a significant increase in pleuromutilins consumption in 2018 though it reduced back down in 2019. Tiamulin, a pleuromutilin, is an antimicrobial agent used mainly in veterinary medicine in many countries. It was introduced in 1979 for the treatment of pulmonary and intestinal infections. In European countries, tiamulin is used for treatment and prevention of swine dysentery (*Brachyspira hyodysenteriae*), treatment of colitis (*Brachyspira pilosicoli*), treatment of ileitis (*Lawsonia intracellularis*) and treatment of enzootic pneumonia (*Mycoplasma hyopneumoniae*). It is also authorised for use in chickens for the treatment and prevention of chronic respiratory disease and airsacculitis caused by *Mycoplasma gallisepticum* and *Mycoplasma synoviae*. Tiamulin is available as medicated premix, oral solution and powder for use in drinking water [34]. Though the reports of resistance development to Tiamulin have been uncommon [35], an increase in minimum inhibitory concentration (MIC) of tiamulin for *Brachyspira isolates* in several countries in Asia, Europe and US, suggesting tiamulin resistance in pigs and in chickens [36].

Findings from this study showed that the total consumption of polymyxins (colistin), based on importation and production data, decreased from 23.5 mg/PCU_Thailand_ in 2018 to 18.6 mg/PCU_Thailand_ in 2019, a result of the recent regulations in 2018 to restrict use of polymyxins. Colistin was commonly added in medicated feed for suckling piglets and nursery pigs for prevention of gastrointestinal tract infections [37]. It has recently become a last-resort antimicrobial for treatment of sepsis and pneumonia caused by extensively drug-resistant Gram-negative bacterial infection in humans. Increased resistance of *K*. *pneumoniae* to colistin was reported in a systematic review [38] and the use of colistin in pigs has been conducive to the development of a plasmid-mediated colistin resistant gene (MCR-1) in commensal *Escherichia coli* from tests on pigs, pork products and humans, which cause global concern [39].

Administering antimicrobials through the oral route for is a common practice and convenient for farm treatment, but poses greater risk to humans due to development of resistant microbes [40]. Oral administration of antimicrobials in the range of 70–90% of total administration has been reported in many studies [23]. However, dosage forms for oral application varied across the globe; for instance, a study in 31 European countries reported oral solution accounting for the majority 57% of total consumption [18] whereas our study showed that medicated premix and oral powder were most common in 2017 and 2018–2019, respectively. The shift from medicated feed to oral powder is the result of the 2018 legal prohibition of inclusion of certain antimicrobials in the medicated feed for disease prevention [27].

Our study identified a gradual increase in animal headcount in Thailand. In response to rapid increases in domestic and international food demands, both the number and the size of intensive large-scale livestock producers have grown significantly. In 2020, about 3.1 million Thai farmers raised about 480 million poultry, 12 million pigs, 7 million cattle, and 1.2 million buffalos [41].

This study includes layer chicken and ducks in the consumption estimates. Thai FDA registered certain veterinary antimicrobials for the use in layer chicken and ducks [42, 43], with evidence of maximum residue limit and withdrawal period prior to approval. The Bureau of Food of the Thai FDA also analyses the level of antibiotic residues from market and supermarket samples in line with Codex Alimentarius guideline [44].

### Limitations of this study

This study uncovers some limitations. The consumption estimated from the volume of imported, exported and locally produced antimicrobials cannot be disaggregated by animal species as many veterinary antimicrobials are approved by Thai FDA for use in multiple species and lack of data granularity. Also, this study cannot estimate the volume of possible extra labels or illegal use. In European countries, data were submitted by MAHs such as pharmaceutical companies licensed for importation and manufacture, wholesalers and feed mills. They submitted the sales or prescription data of veterinary antimicrobials to the European Medicines Agency. Most countries collect sales data, except a few countries including Bulgaria, Hungary, Malta, Netherlands, and Spain where sales data is based on the national law on animal drug control [18]. Sales data would be closer to the actual use data compared to importation and production data due to free of stock effect, but Thailand regulations do not require pharmaceutical companies to submit sales data.

Sales data alone does not allow estimate consumption by animal species or stage of animal production. However, antimicrobial use data collated by national surveillance systems can be used to measure antimicrobial use by species or stage of production. It can also assess the impacts of interventions, as performed in some European countries [45, 46]. Analysing data on antimicrobial use in conjunction with AMR profiles in specific settings allows the interpretation of the relationship between antimicrobial use and AMR, and it helps better implementation of antimicrobial stewardship [47, 48]. For example, several studies demonstrated a robust association between multidrug-resistant *E*. *coli* and the parenteral use of aminoglycosides in turkeys [49].

This study provides evidence for macro-level monitoring of total consumption in animal sector, though lack of specificity on use such as indications, duration and route of administration by animal species for which farm level data collection can serve this purpose. A few steps were taken in Thailand; DLD has established a system to collect antimicrobial use data from medicated feed; this facilitated monitoring of antimicrobial consumption through medicated feed by key species since 2019. However, the data on the doses, durations, route and indications were not available. In some studies, quantitative and qualitative data were collected by using a survey form or interviews with veterinarians to identify antimicrobial class, active ingredient, and route of administration [33, 36, 50].

Moreover, national importation and production cannot capture the use of antimicrobial by different animal production system, notably backyard and smallholder farm versus large integrated production. Few studies raised concern on the inappropriate antimicrobial use in backyard systems such as use of over-the-counter veterinary medicines available from retail outlets and lack of veterinary oversight [51]. A study from Thailand showed a low-level understanding about proper use of antibiotics among smallholder pig farmers [52].

In addition, in this study we assumed that the total antimicrobial consumption is equal to the production and importation minus total exports. Although there are variations in annual stock, in an efficient pharmaceutical market, the stock level should be constant in each year. This assumption may not hold true in every instance as the level of stock is determined by price and demand and availability of products.

Also, given that Thai experts assumed standing weight as average weight (AW) for certain animals such as broiler breeders, layer breeders, and laying hens as no AW data was available from ESVAC, the PCU in this study and European countries is determined slightly differently and should be subject to careful interpretation, especially when comparing it between countries. The WOAH suggests its member countries report milligram of antimicrobials use adjusted for kilogram of animal biomass [3]. The biomass calculation accommodates species differences in body weight and production cycle using both census and slaughter data [53]. For example, in pigs, biomass was calculated by summing the expected biomass of fattening pigs slaughtered in a country in one year and of pigs retained for breeding purpose. The formula is (live weight x number of animals slaughtered) + (census population x sow weight x 0.09). In chickens, biomass was calculated by multiplying the live weight of the chicken by the number of chickens slaughtered. However, in Thailand, it is in Thailand, data on slaughtered animals is not available which limits the ability to calculate biomass in this way. There is ongoing discussion among stakeholders to produce Thai biomass data for different animal species.

## Conclusion

In conclusion, this study provides the first data on a three-year period and related trends of antimicrobial consumption in food producing animals in Thailand. There was a significant decrease of consumption between 2017 and 2019, especially of antimicrobials under DLD restrictions. The restricted antimicrobials were replaced by non-CIA group antimicrobials such as pleuromutilins. The government should continue to monitor and minimise the use of antimicrobial consumption particularly of the CIA category.

This study provides valuable baseline data of monitoring progress and impact of policies and strategies for the containment of AMR and strengths antimicrobial stewardship. In the context of increased prevalence of AMR in veterinary practices, national monitoring of antimicrobial consumption and resistance should be integrated and synthesised to inform policies for prudent use of antimicrobial. The ongoing DLD efforts in monitoring antimicrobial use by animal species can guide specific policy interventions for different species.

## Supporting information

S1 Data Summary data 2017–2019. (XLSX)

S2 Data Animal population 2017–2019. (XLSX)
